# Validation of the Italian Version of the Educational Needs Assessment Tool in Rheumatoid Arthritis Patients and Factors Associated with Educational Needs

**DOI:** 10.3390/jpm10040150

**Published:** 2020-10-01

**Authors:** Marta Favero, Francesca Ometto, Fausto Salaffi, Elisa Belluzzi, Augusta Ortolan, Mariagrazia Lorenzin, Mara Felicetti, Leonardo Punzi, Mwidimi Ndosi, Roberta Ramonda

**Affiliations:** 1 Rheumatology Unit, Department of Medicine (DIMED), University of Padova, 35128 Padova, Italy; faveromarta@gmail.com (M.F.); Francesca.ometto@unipd.it (F.O.); elisa.belluzzi@gmail.com (E.B.); augusta.ortolan@yahoo.it (A.O.); mariagrazialorenzin@libero.it (M.L.); mara.felicetti@gmail.com (M.F.); 2 Rheumatological Clinic, Ospedale Carlo Urbani, Università Politecnica delle Marche, 60035 Jesi, Italy; fausto.salaffi@gmail.com; 3 Centre for Gout and Metabolic Bone and Joint Diseases, Rheumatology, SS Giovanni and Paolo Hospital, 30122 Venice, Italy; leonardo.punzi@unipd.it; 4 Department of Nursing and Midwifery, University of the West of England, Bristol BS16 1DD, UK; mwidimi.ndosi@uwe.ac.uk; 5 Academic Rheumatology Unit, University Hospitals Bristol, Bristol BS2 8HW, UK

**Keywords:** rheumatoid arthritis, educational needs assessment tool

## Abstract

The educational needs assessment tool (ENAT) is a seven-domain questionnaire assessing the educational needs (EN) of patients with rheumatoid arthritis (RA). The aim of this study was to validate the Italian version of the ENAT and to identify factors associated with EN in people with RA. The original English ENAT version was translated into Italian according to Beaton’s method and subjected to Rasch analysis for validity testing. Socio-demographic and clinical variables were tested for associations with the ENAT domain scores using a multivariable linear regression model. The ENAT translated well into Italian and retained its construct validity. Some adjustments were needed when pooling the Italian and English datasets. The overall score of the ENAT had a high median: 82.8 (interquartile range (IQR): 57.5 to 100) i.e., 72.4% of the maximum score. The highest score was observed in the domain “Arthritis process” and the lowest was in “Support systems”. Only gender was independently associated with EN (females having higher EN than males). The Italian ENAT is feasible for the use in the clinical setting and may help the health care practitioners to tailor educational interventions for RA patients. The characteristics of the patients, particularly female gender, may be associated with higher EN.

## 1. Introduction

Patient education is an important part of rheumatoid arthritis (RA) management and should complement clinical care. Several studies clearly demonstrate that patient education improves treatment adherence and clinical outcomes, especially in the short term [1,2,3,4]. Although most national and international guidelines recommend that patient education is adequately addressed by health care practitioners, [5,6,7,8] only a few studies have been carried out to deepen the knowledge on what the educational needs are, as perceived by RA patients.

To date, the Educational Needs Assessment Tool (ENAT) is the only questionnaire to assess the educational needs of RA patients. It was developed by patients and practitioners in the United Kingdom (UK). [9] The questionnaire is composed of 39 items, grouped into seven domains: managing pain (six items), movement (five items), feelings (four items), arthritis process (seven items), treatments from health professionals (seven items), self-help measures (six items) and, support systems (four items). Each item has a Likert-type answering scale ranging between zero and four: “not at all important”, “a little important”, “fairly important”, “very important” and “extremely important”. The sum of the score of each item gives the total score, ranging from zero to 156. The ENAT showed a good test–retests reliability [9]. Furthermore, it showed a good fit to the Rasch model which confirms a good construct validity, unidimensionality of the scale and invariance to gender, age, educational background and disease duration [10]. Rasch-transformed scores are available to allow normalisation of the scores and facilitate the analysis of potential associations with other patient and disease characteristics [11,12,13]. Additionally, percentage scores of the total score and of the score of each domain have been used to compare the results of each domain [14].

The ENAT was adapted and validated in nine European languages for RA [11,15]. The cross-cultural validation enabled the comparison of educational needs of different countries and the possibility to pool datasets from different countries (Austria–Germany, Finland, The Netherlands, Norway, Poland, Portugal, Spain, and Sweden). The ENAT has been used also to assess educational needs in other rheumatic diseases other than RA such as systemic sclerosis, spondylarthritis, systemic lupus erythematosus, psoriatic arthritis, fibromyalgia, and osteoarthritis [14,16,17,18,19,20,21]. Only a few analyses have been conducted to assess the association between the ENAT results and patients and disease characteristics and only one is available with adjustment for potential confounders [22].

The objective of this study was to validate an Italian version of the ENAT in RA patients. Then, a cross-sectional analysis was conducted to investigate what socio-demographic and clinical factors are associated with the educational needs of RA patients.

## 2. Materials and Methods

### 2.1. Study Design

The study was conducted in two phases. The first phase comprised the cross-cultural adaptation of the English ENAT into Italian and validation of the adapted tool in patients with RA. The second phase investigated what socio-demographic and clinical variables were associated with the educational needs of the patients as assessed by the Italian ENAT.

### 2.2. Patients

Patients were recruited from the outpatient clinic of Padova University Hospital. The inclusion criteria were: (a) aged 18 or above; (b) diagnosis of RA according to the American College of Rheumatology/European League Against Rheumatism (ACR/EULAR) classification criteria [23]; and (c) ability to complete the questionnaire unaided. Exclusion criteria were: (a) inability to read or write; and (b) concomitant rheumatic disease other than RA. All participants provided a written informed consent before inclusion in the study. The study was carried out in accordance with the ethical standards of the Declaration of Helsinki (1983) and was approved by the Local Ethical Committee for the Clinical Trials of the Province of Padova (Prot. n° 2978P).

### 2.3. Phase I. Cross-Cultural Adaptation and Validation of ENAT into Italian

Like other validations, the ENAT was translated into Italian according to Beaton’s cross-cultural adaptation method [24]. The adaptation process included 5 steps: (1) initial translation from English to Italian; (2) synthesis of the translations; (3) back (blind) translation into English; (4) review of an expert committee which decides on conceptual equivalence between the source and target versions; and (5) test of the pre-final version in 30 RA patients [24]. The expert committee included a methodologist, 3 health professionals, all the translators, a moderator of the translations and a member of the ENAT development group. After the expert committee deemed that an accurate Italian version of ENAT was achieved, it was completed by a sample of consecutive patients fulfilling the study criteria and, then it was subjected to validation.

#### Statistical Analysis

Following data cleaning, the ENAT data were assessed for validity using the Rasch measurement model [25]. Fit to the Rasch model implies construct validity, reliability and statistical sufficiency of the total score from the scale. Model fit is the extent to which observed item response data (standardised residuals) accord with specifications of the Rasch model. This is tested using a set of fit statistics with the null hypothesis of no significant difference between the observed values and those expected by the model. The reliability of the scale was tested within Rasch analysis using Person-Separation-Index (PSI) which is an estimate of internal consistency evaluating discrimination between people with different levels of educational needs.

Cross-cultural invariance was tested by pooling the Italian dataset with the original (English) data, then differential item functioning (DIF) by culture was tested [11,26]. Like in previous studies, a conversion chart was calibrated to help transform the raw ENAT scores, which are at ordinal level into interval-level (Rasch-transformed scores) [11,12,13]. Such transformation allows the use of ENAT scores in parametric statistics and more flexible statistical analyses alongside other outcome measures. Bonferroni adjustment was applied in all tests (i.e., *p* = 0.05/number of tests carried out) to avoid type I errors resulting from to multiple testing [27].

### 2.4. Phase II. Cross-Sectional Analysis: Factors Associated with the Italian ENAT Score

To evaluate factors associated with educational needs, the following explanatory variables were assessed: 5 socio-demographic variables (sex, age, school leaving age, social status, employment), 10 clinical variables (disease duration, clinical assessments, smoking status, comorbidity) and 7 patient-reported outcomes (PROs). PROs were: question regarding the will to receive information (yes/no answer) and the amount (Likert scale, 0 = “very little” and 4 = “a lot”); health assessment questionnaire (HAQ) (ranging from 1 to 3); a question if the patient was experiencing a disease flare(yes/no answer); global “health” question (Likert scale, 1 = “very bad” and 4 = “very good”); and disease “severity” question (Likert scale, with 1 = “not severe” and 4 = “very severe”). PROs were completed at the same time of the ENAT. To avoid collinearity in multivariable analysis, the 28-joint disease activity score (DAS28) was not considered in favour of its components which were deemed more informative as potential predictors of the educational needs (i.e., erythrocyte sedimentation rate, C-reactive protein, patient activity assessment on a visual analogic scale (patient-VAS), swollen joint count and tender joint count). The patient reported outcome, HAQ, was also included in the analysis of predictors of the ENAT being a measure of both severity and disability of RA. Employment status was considered as the following categories: “housewife”, “retired” and “full-time employment”, being the three most represented categories. Association of the ENAT with other PROs was analysed separately and not included in the multivariable analysis of predictors of the ENAT results.

#### Statistical Analysis

Scores expressed as percentages were computed (=score/maximum score × 100) to allow for comparison between domains and were used to compare the responses of the different domains. Rasch-transformed scores of total ENAT and of each domain were used to test the association with other variables. Normality was explored according to the Shapiro-Wilk test. No variable was found to be normally distributed. All continuous variables were expressed as medians with the corresponding interquartile range (IQR). Associations of scores with continuous variables were tested with Spearman’s correlation. Associations of scores with categorical variables were tested with the Kruskal–Wallis test. Post-hoc tests were conducted with the Kruskal–Wallis test with Bonferroni adjustment. To test independent associations with the ENAT results, a multivariable linear regression model was used. Variables included were those showing an association with *p* < 0.10 in univariable analyses. A variance inflation factor of 2 was used as a cut-off to exclude variables because of collinearity. Regression coefficients beta-estimates are presented with 95% confidence intervals (CI). All analyses were performed with IBM SPSS Version 24.0. Armonk, NY, USA: IBM Corp.

## 3. Results

### 3.1. Patients Characteristics

One hundred and fifty consecutive patients admitted to our rheumatology unit and fulfilling the study criteria were recruited. Forty-three patients were excluded because of inability to read or write or because of a concomitant rheumatic disease other than RA. The first 30 enrolled patients were included in the field testing. The characteristics of the population are reported in Table 1. Data of the Italian and English [10] cohorts used for the ENAT cross-cultural validation and adaptation are reported in Table S1, Supplementary Materials.

### 3.2. Phase I. Cross-Cultural Adaptation and Validation

The Italian ENATs completed by further 120 patients were collected and a first observation emerged. The five response categories: “not at all important”, “a little important”, “fairly important”, “very important” and “extremely important” did not work as intended. There were disordered thresholds across a few items where patients found it difficult in discriminating between “a little important” and “fairly important”. This did not affect the “item fit” to the model (Table S2, Supplementary Materials). However, the overall scale fit was affected due to significant item–item correlations (r > 0.3) [28] which violated the Rasch model assumption of local independence of items. Then, all locally dependent items were grouped into their respective domains, creating a seven testlet-scale, each testlet (or subscale) demonstrating a good fit to the model (Table S3, Supplementary Materials). Table 2 presents the fit statistics of the whole scale, first as a 39-item scale (Analysis 1) and then as a seven-testlet scale (Analysis 2). For the Italian ENAT, the preliminary fit statistics of the 39-items (Table 2, Italy, Analysis 1) demonstrate a significant deviation from the Rasch model χ^2^(df) = 84.341 (39), *p* < 0.01. After grouping all items into testlets (subscales), a second analysis (Table 2, Italy, Analysis 2), revealed a good fit to the Rasch model, χ^2^(df) = 3.360 (7), *p* = 0.850 and internal consistency (Person separation index) = 0.878.

**Figure 1:**
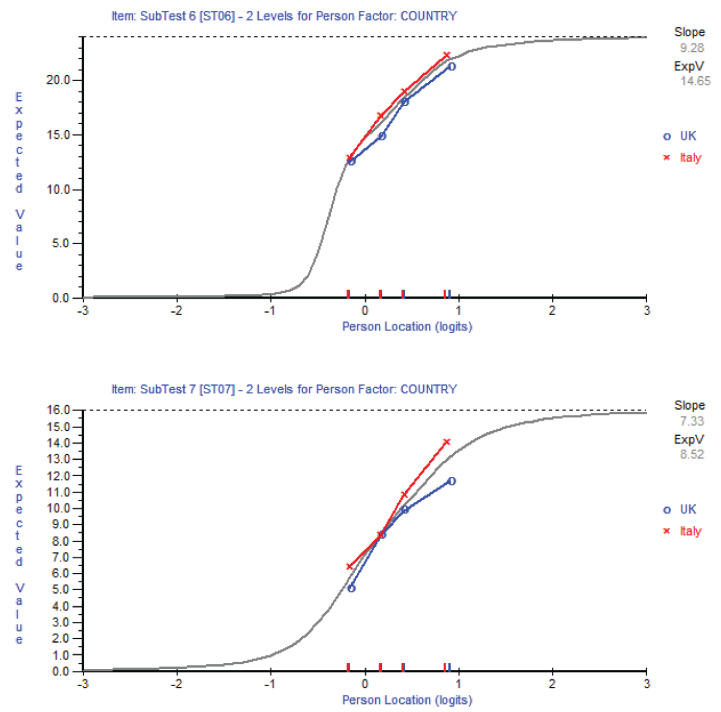
Cross-cultural differential item functioning.

Pooling the Italian data with the original English data, revealed cross-cultural DIF (Figure 1) and adjustments were necessary for two subscales: “Self-help measures” and “Support systems”.

A conversion chart was calibrated to help transform the raw ENAT data (which are at ordinal level), into Rasch-transformed data (Table 3). The conversion chart also has adjusted scores for the “Self-help measures” and “Support systems”, which accounts for non-equivalence of measurements when comparing the data from Italy and the UK (Table 3).

### 3.3. The Scores of the Italian ENAT

Response rates, raw scores, Rasch-transformed scores and scores expressed as percentages of the ENAT and domains are reported in Table 4. Among the collected questionnaires, 107 (89.2%) were fully completed (Table 4).

Most of the patients were willing to receive information (84.2% answered “yes” to the specific question). Furthermore, 67.5% of the patients wanted to receive “all the information they needed to know” (the highest score on the four-point Likert answering scale). The response rate was good, being between 94.2 and 100% for each domain. The domain with the least response rate was “arthritis process” while all the patients answered the “support systems” domain. The overall score of the ENAT was high: median: 82.8 (IQR 57.5 to 100), which is 72.4% of the maximum score. The highest score for the educational needs was observed in the domain “arthritis process”, being 82.1% (raw value 23), and the lowest were “support systems” and “managing pain”: 62.5% (raw value 10) and 66.7% (raw value 16), respectively (Table 2).

### 3.4. Phase II. Factors Associated with Educational Needs

The associations of the scores of each domain with continuous variables are reported in Table S4, Supplementary Materials, and with categorical variables in Table S5, Supplementary Materials. Table 5 reports the multivariable regression models for the analysis of factors independently associated with the score of the total ENAT and of each domain. All models were significant but with a low R square (ranging from 0.095 to 0.192), indicating that the variables included in the models predicted only a little of the variability of the scores. The total ENAT score was independently associated only with gender with female gender being associated with higher educational needs than the male gender. Female gender was also associated with higher scores in “managing pain”, “feelings”, “treatment from health-professionals” and “self-help measures” domains. A high HAQ, indicating a functional impairment, was associated with high scores in the “movement” domain and a high number of swollen joint count, being a sign of active inflammatory disease, was independently associated with the “arthritis process” domain. Patient-VAS, representing the patient’s assessment of the disease, was independently associated with “self-help measures” and “support systems” domains with worse patient-VAS being associated with higher educational needs in “self-help measures” and “support systems” domains (Table 5).

#### Association of the Italian ENAT Scores with Patient-Reported Outcomes

The associations of the scores of each domain and patient-reported variables (continuous and categorical) are reported in Tables S4 and S5, Supplementary Materials. As expected, patients who were willing to receive information showed significantly higher scores in the total Italian ENAT score and all domains except for “feelings”, “self-help measures” and “support systems”. HAQ score was positively correlated with total score and with the score of all domains except for “managing pain” and “support systems”. Nevertheless, the correlation was in all cases fair. Patients reporting a concomitant flare scale showed significant association only with “treatment from health professionals”. The “health” question was significantly associated with all domains except from “managing pain”, “feelings” and “arthritis process”. Higher ENAT scores were observed in patients reporting worse health scores. The “severity” question showed no significant association with any of the domain (almost significant only for “movement”).

## 4. Discussion

This is the first study to explore the educational needs of patients in Italy by the means of a validated tool, the Italian version of the original ENAT questionnaire.

Rasch analysis allowed correction of the local dependence and cross-cultural bias, thus enabling a robust assessment of educational needs in patients with RA in Italy. Using the cross-cultural adjustments allows comparison of the Italian and the UK data. Furthermore, we found that educational needs of patients with RA vary because of subjective and clinical characteristics. Particularly, female gender was associated with higher educational needs and a more severe disease activity was associated with higher scores in specific domains of the ENAT such as “movement” and “arthritis process”.

Standardised methods for cross-cultural adaptation of outcome measures were used to develop the Italian version of the ENAT. The adaptation into Italian was smooth and the ENAT was well received by patients since 90% completed the entire questionnaire. The “arthritis process” domain was the least completed domain, and “support systems” was completed by all patients which may reflect either a minor comprehension or a lower interest about the specific domains. The high rate of responses confirms the feasibility of the questionnaire and that it is suitable for the use in the clinical setting. Response rates in some studies testing ENAT in large cohorts of patients affected by rheumatic conditions [14] were not as high as in our study, although very good response rates are reported also in other studies [18,29,30]. We observed good responses to the initial question about the willingness to receive information, with two thirds of the patients willing to receive as much information as possible, which is very similar to previous reports in RA [14,18]. As expected, the willingness to receive more information was associated with higher scores in all domains of the ENAT.

The total ENAT Rasch-transformed score was high (82.8), similar to other studies [14,18]. It has to be taken into account that comparison with other studies is not straightforward since most studies presented only raw scores. Scores presented as percentages allowed the comparison between domains in our analysis with highest score for “arthritis process” (82.1% of the maximum score) and lowest for “managing pain” and “support systems” which was between 60–70% of the maximum score. It is noteworthy that “arthritis process” was the least answered domain while “support systems” was the most answered. This may be explained by a little comprehension of contents of the “arthritis process” testlets but high interest of the patients who understood and answered to the domain. On the other hand, questions in “support systems” may be more comprehensible but the patients have a lower interest since their needs about this topic (mainly patients’ organisations and improvement of health care arrangements) may be already addressing it. Studies conducted among Polish and Dutch RA patients found similar results except from the domain “support systems” which received higher scores in Dutch patients [14]. Differences with other countries are expected, especially regarding “support systems” which also involves the satisfaction of the patient regarding the health care system of their country.

The major determinant of the ENAT score in this study was found to be gender, with female patients presenting the highest educational needs in almost all domains. Such association has already been found in other cohorts including patients affected by osteoarthritis and spondylarthritis, and similar to our findings, especially in some domains such as “managing pain”, “feelings”, “treatment from health professionals”, “self-help measures” and “movement” [14,18,20]. A study evaluating educational needs in female patients with a recent pregnancy, revealed the highest scores in ENAT, further confirming that females are more willing to receive information compared to males [31]. RA female patients have a different perception of the disease and of pain compared to males, usually presenting with worse subjective measures of the disease [32]. Our study further supports the evidence that a gender approach might be more suitable in the management of RA.

Disease severity appeared to affect the educational needs of patients to a lesser extent than gender. This might be explained that most of the patients had a low disease activity as assessed by DAS28. Nonetheless, a higher HAQ, which reflects impairment of function, disability and possibly a longer disease duration, was independently associated with higher scores in the “movement” domain. Similar findings may be found in Dutch and Polish RA patients, in which “movement” was associated with disease duration and severity. Of note, also a higher number of swollen joints, which is a sign of active disease, was associated with higher educational needs in the “arthritis process” domain which seems consistent with the fact that patients experiencing high activity of the disease are willing to better understand also the biological processes underlying RA. A worse patient-VAS was associated with higher scores in “self-help measures” and “Support systems”. Unsatisfactory disease control, as perceived by the patient, is associated with the need for improvement in the health care system and may also lead to an interest in alternative treatment approaches, which are mentioned in “self-help measures”.

Differently from previous studies, no significant association was found with age, education or employment [14,18]. Indeed, in the study cohort, higher scores were observed among unemployed or part-time patients in “treatment from health professionals”, “self-help measures” and “support systems” domains but this result was not confirmed after adjustment for potential confounders.

Similarities with educational needs in other countries were not expected. Firstly, only a few studies which include the analyses of factors associated with educational needs in RA patients are available, and most of them did not adjust the results for potential confounders. Secondly, every country has peculiar social characteristics and importantly, different health care systems. Since the education of the patient is primarily delivered by health care practitioners, we believe that lower educational needs may be seen in the cohorts in which patients have a better health care system.

The ENAT score showed a fair association with the Health question and with HAQ. This supports the consistency of the results, nonetheless, a fair correlation further confirms that the ENAT addresses educational needs and not the patient’s assessment of disease activity.

The main limit of the study is that the sample was not representative of the entire RA population in Italy. Firstly, the study was monocentric, thus, the educational needs of Italian RA patients from other areas may be affected by different characteristics. Secondly, about 30% of the patients had to be excluded because of an inability to complete the questionnaires. The patients not included in the analysis were mostly elderly or unable to speak Italian. These groups of patients also have educational needs which may be very specific and should be addressed separately. Nonetheless, the study provided a validated tool to measure educational needs which may be used by every health care practitioner in the field of rheumatology. Furthermore, this is the first report about the educational needs of RA patients in Italy to date.

Patient’s education delivers benefits in the health status of RA patients. Nonetheless, to be successful, education should be customised on the basis of patient needs [33]. The ENAT was developed to help in this purpose, that is to enable patient to identify and prioritise their educational needs. Based on the ENAT results, practitioners are able to systematically assess the priorities, needs and expectations of patients affected by RA and thus provide appropriate information and possibly also tailor treatment decision.

## 5. Conclusions

In conclusion, this study has demonstrated that the Italian ENAT retained the original construct validity after cross-cultural adaptation although, cross-cultural adjustments are required if the Italian data are pooled or compared with the UK data. Educational initiatives could be preceded by the administration of this simple-to-complete questionnaire which may be used in the clinical practice. Health care professionals should bear in mind that female RA patients are more likely to need information, and that, based on existing health care systems and disease severity the patients may have slightly different educational interests. Importantly, further studies should be conducted to assess the success of educational intervention based on needs identified through the ENAT questionnaire. Furthermore, given the importance of tele-medicine, a mobile version of the ENAT questionnaire may also be spread among Italian RA patients in order to reach a larger number of patients who will provide useful insight on their educational needs.

## Figures and Tables

**Table 1:** Characteristics of the population.

Characteristic	
Females, n (%)	97 (80.8)
Age, years, median (IQR)	55 (43.5;64)
Disease Duration, years, median (IQR)	15 (9;21)
School leaving age, years, median (IQR)	17 (14;19)
Smoking status	
Non smoker, n (%)	41 (43.2)
Smoker, n (%)	17 (14.2)
Former smoker, n (%)	24 (20)
Social status, n (%)	
Married/domestic partner, n (%)	89 (74.2)
Widow/widower, n (%)	1 (0.8)
Divorced, n (%)	9 (7.5)
Not married, n (%)	20 (16.7)
Employment	
Full-time, n (%)	35 (29.2)
Housewife, n (%)	35 (29.2)
Retired, n (%)	28 (23.3)
Part-time employement, n (%)	9 (7.5)
Invalid, n (%)	1 (0.8)
Unemployed, n (%)	5 (4.2)
Student, n (%)	2 (1.7)
Other, n (%)	2 (1.7)
Concomitant comorbidity, n (%)	49 (40.8)
CRP, median (IQR), mg/dL	2.9 (2.1;5.5)
ESR, median (IQR), mm/h	16 (6;29)
Tender joint count, median (IQR)	1.5 (0;4)
Swollen joint count, median (IQR)	0 (0;2)
Patient—VAS, median (IQR), mm	50 (20;62.5)
28-joint disease activity score (CRP), median (IQR)	2.8 (1.9;3.6)
28-joint disease activity score (ESR), median (IQR)	3.3 (2.2;4.2)
Patient-reported outcomes	
HAQ, median (IQR)	0.8 (0.4;1.4)
Other person’s help, activities number, median (IQR)	1 (0;2)
Tools used, activities number, median (IQR)	0 (0;1)
Willing to receive information (yes, %)	101 (84.2)
How much information	
1	3 (2.5)
2	14 (11.7)
3	21 (17.5)
4	81 (67.5)
Disease Flares	
Yes	47 (39.2)
No	70 (58.3)
Health Question	
1	6 (5)
2	35 (29.2)
3	47 (39.2)
4	32 (26.7)
Severity Question	
1	14 (11.7)
2	48 (40)
3	44 (36.7)
4	13 (10.8)

IQR: interquartile range, CRP: C-reactive protein, ESR: erythrocyte sedimentation rate, VAS: Visual Analogic Score, HAQ: Heath assessment questionnaire.

**Table 2:** Summary of the results of the Rasch analysis.

		Item Fit Residual		Person Fit Residual		Chi Square Interaction		PSI		Proportion of Independent *t*-Tests (95%CI)
	Analysis	Mean	SD	Mean	SD	Value (df)	*p*		N	
UK	Analysis 1 (initial)	0.344	1.685	−0.267	2.011	71.289 (39)	0.001	0.935	119	
	Analysis 2 (Subtest)	0.541	0.699	−0.308	1.168	7.116 (7)	0.417	0.838	119	0.065 (0.026, 0.103)
Italy	Analysis 1 (initial)	0.407	1.348	−0.263	2.066	84.341 (39)	<0.01	0.947	117	
	Analysis 2 (Subtest)	0.373	0.696	−0.290	1.068	3.360 (7)	0.850	0.878	117	0.084 (0.045, 0.123)
Pooled	Analysis 1 (initial)	0.501	2.086	−0.351	2.144	268.864 (117)	<0.01	0.939	236	
	Analysis 2 (Subtest)	0.441	0.861	−0.347	1.143	13.891 (21)	0.874	0.848	236	0.066 (0.039, 0.094)
Adjusted	Analysis (7-subscales)	0.392	0.968	−0.337	1.330	19.843 (27)	0.837	0.855	236	
Perfect fit to the model		0	1	0	1		>0.05	>0.7		Lower 95%CI < 0.05)

SD: standard deviation, df: degrees of freedom, PSI: Person-Separation-Index, CI: confidence of interval.

**Table 3:** Conversion chart from raw score to Rasch transformed scores.

Raw Score	Rasch-Transformed Scores								
	Managing Pain	Movement	Feelings	Arthritis Process	Treatments from Health Professionals	UK Self-Help Measures	Italy Self-Help Measures	UK Support Systems	Italy Support Systems
0.0	0.0	0.0	0.0	0.0	0.0	0.0	0.0	0.0	0.0
1.0	1.6	1.1	1.3	0.1	1.0	0.5	0.3	1.3	1.2
2.0	2.7	2.0	2.2	0.3	1.4	0.9	0.7	2.3	2.1
3.0	3.4	2.7	3.0	0.7	1.7	1.2	1.0	3.0	2.8
4.0	4.1	3.2	3.6	0.9	1.9	1.5	1.0	3.7	3.4
5.0	4.7	3.7	4.2	1.1	2.2	1.8	2.3	4.3	4.0
6.0	5.2	4.2	4.7	1.5	2.6	2.1	1.8	4.9	4.6
7.0	5.8	4.7	5.3	2.0	2.8	2.3	2.0	5.7	5.3
8.0	6.4	5.1	5.9	2.0	3.0	2.7	2.3	6.5	6.2
9.0	7.0	5.7	6.6	2.0	3.4	2.9	2.6	7.5	7.2
10.0	7.7	6.2	7.4	2.3	3.6	3.2	2.9	8.6	8.4
11.0	8.6	6.8	8.3	2.5	3.8	3.6	3.1	9.6	9.4
12.0	9.5	7.6	9.2	2.8	4.0	4.1	3.5	10.5	10.4
13.0	10.5	8.5	10.3	3.1	4.4	4.7	3.8	11.5	11.4
14.0	11.5	9.5	11.7	3.4	4.7	5.8	4.3	12.6	12.6
15.0	12.5	10.6	13.5	3.7	5.1	8.6	4.9	14.1	14.0
16.0	13.5	11.8	16.0	4.1	5.5	10.5	7.1	16.0	16.0
17.0	14.5	13.1		4.6	6.2	12.0	12.2		
18.0	15.4	14.7		5.6	7.5	13.4	13.7		
19.0	16.4	16.9		8.8	10.0	14.8	15.0		
20.0	17.4	20.0		10.6	11.9	16.0	16.3		
21.0	18.4			12.4	13.4	17.4	17.7		
22.0	19.7			13.9	14.8	19.0	19.1		
23.0	21.5			15.4	16.2	21.1	21.2		
24.0	24.0			17.0	17.7	24.0	24.0		
25.0				18.8	19.3				
26.0				20.8	21.2				
27.0				23.8	24.0				
28.0				28.0	28.0				

**Table 4:** The Italian educational needs assessment tool (ENAT) scores.

	Total ENAT	Managing Pain	Movement	Feelings	Arthritis Process	Treatments from Health Professionals	Self-Help Measures	Support Systems
Answered items, n (%)	107 (89.2)	114 (95)	117 (97.5)	119 (99.2)	113 (94.2)	119 (99.2)	118 (98.3)	120 (100)
Raw scores, median (IQR)	102 (83.5;114.5)	16 (12;18)	14 (10;16)	11 (8;13)	23 (20;26)	21 (17;24)	17.5 (14;20)	10 (7;12)
Rasch-transformed scores, median (IQR)	82.8 (57.5;100)	13.5 (9.5;15.4)	9.5 (6.2;11.8)	8.3 (5.9;10.3)	15.4 (10.6;20.8)	13.4 (6.2;17.7)	13 (4.3;16.3)	8.4 (5.3;10.4)
Percentage scores, median (IQR)	72.4 (59;80.4)	66.7 (50;75)	70 (50;80)	68.8 (50;81.3)	82.1 (71.4;92.9)	75 (60.7;85.7)	72.9 (58.3;83.3)	62.5 (43.8;75)

IQR: interquartile range.

**Table 5:** Multivariable Linear regression, analysis for the total Italian ENAT score and for each ENAT domain.

	OR (95% C.I.)	*p* Value
Total ENAT		
Female	18.53 (2.19;34.88)	0.027 *
ESR, per 10-mm increase	3.04 (−1.22;7.3)	0.160
Patient—VAS, per 10-unit increase	2.47 (−0.38;5.32)	0.089
HAQ, per unit increase	6.35 (−3.45;16.15)	0.201
Model Significance 0.001; R square 0.183		
Managing pain		
Female	2.16 (0.05;4.27)	0.045 *
ESR, per 10-mm increase	0.4 (−0.12;0.91)	0.131
Patient-VAS, per 10-unit increase	0.3 (−0.02;0.61)	0.068
Model Significance 0.016; R Square 0.099		
Movement		
Female	2.08 (−0.18;4.33)	0.071
Patient-VAS, per 10-unit increase	0.24 (−0.15;0.63)	0.228
HAQ, per unit increase	2.24 (0.86;3.62)	0.002 *
Model Significance <0.001; R Square 0.192		
Feelings		
Female	2.24 (0.26;4.22)	0.027 *
Housewife	0.57 (−1.2;2.34)	0.524
ESR, per 10-mm increase	0.25 (−0.22;0.71)	0.291
Patient-VAS, per 10-unit increase	0.02 (−0.31;0.35)	0.910
HAQ, per unit increase	1.11 (−0.08;2.3)	0.068
Model Significance 0.008; R Square 0.143		
Treatments from health professionals		
Female	3.78 (0.17;7.4)	0.040 *
School leaving age, per year increase	−0.01 (−0.03;0)	0.109
Full-time employment	−1.31 (−4.58;1.96)	0.430
Patient-VAS, per 10-unit increase	0.43 (−0.2;1.05)	0.180
HAQ, per unit increase	1 (−1.3;3.3)	0.391
Model significance 0.015; R Square 0.118		
Arthritis process		
Female	2.04 (−1.63;5.71)	0.274
Swollen joint count	−0.51 (−0.96;−0.07)	0.024 *
Patient-VAS, per 10-unit increase	0.51 (−0.12;1.15)	0.109
HAQ, per unit increase	1.4 (−0.88;3.68)	0.227
Model significance 0.018; R Square 0.128		
Self-help measures		
Female	3.42 (0.17;6.67)	0.039 *
Patient-VAS, per 10-unit increase	0.65 (0.07;1.23)	0.028 *
HAQ, per unit increase	0.09 (−1.96;2.14)	0.93
Model significance 0.010; R Square 0.095		
Support systems		
Female	1.74 (−0.09;3.56)	0.062
School leaving age, per year increase	0 (−0.01;0.01)	0.911
Retired	−1.55 (−3.25;0.15)	0.073
ESR, per 10-mm increase	0.14 (−0.3;0.59)	0.521
Patient-VAS, per 10-unit increase	0.35 (0.03;0.67)	0.032 *
HAQ, per unit increase	0.38 (−0.77;1.53)	0.511
Model significance 0.005; R Square 0.167		

OR: odds ratio, CI: confidence of interval, ESR: Erythrocyte sedimentation rate, VAS: Visual Analogic Score, HAQ: heath assessment questionnaire. *significant associations (*p* value <0.05).

## Data Availability

All data relevant to the study are included in the article or uploaded as supplementary information. Any other data are available from the corresponding author. The ENAT is free to use for educational and non-profit research purposes but permission is required from the University of Leeds who owns the copyright of the instrument. To obtain copies of the original and translated versions of the ENAT and permission, please contact the Psychometric Laboratory at the University of Leeds, Leeds Institute of Rheumatic and Musculoskeletal Medicine, email: RehabMed@leeds.ac.uk.

## Supplementary Materials

The following are available online at https://www.mdpi.com/2075-4426/10/4/150/s1, Table S1: Patient characteristics in the Italy and UK datasets, Table S2: Item fit statistics, individual items, Italian (I- ENAT), Table S3: Item fit statistics for the 7 domains, Italian ENAT (I-ENAT), Table S4: Association of Rasch-transformed scores of the I-ENAT with continuous variables (Spearman’s correlation), Table S5: Association of Rasch-transformed I-ENAT scores with categorical variables (Kruskal-Wallis test and post-hoc tests), all values reported as medians and interquartile range of the I-ENAT scores.
