# Supplementary material for: Validation of the Italian Version of the Educational Needs Assessment Tool in Rheumatoid Arthritis Patients and Factors Associated with Educational Needs
Source: J Pers Med. 2020 Oct 1;10(4):150. doi: 10.3390/jpm10040150 (PMC7712017; doi:10.3390/jpm10040150)
Supplement: Supplementary file 1 [file jpm-10-00150-s001.pdf]

SUPPLEMENTARY MATERIAL

Supplementary Table 1. Patient characteristics in the Italy and UK datasets.

	**Italy**	**UK**
Sample size	120	125
Women, n (%)	97 (80.8)	99 (79.2)
Age, years, mean (SD)	52.95 (14.83)	55.90 (13.16)
Disease duration, years, mean (SD)	15.49 (9.59)	13.97 (9.38)
Basic education, n (%)	58 (48.3)	105 (86.1)

SD standard deviation.

Supplementary Table 2. Item fit statistics, individual items, Italian (I- ENAT).

	Item	Fit Residuals	Degrees of freedom	Chi Square	Probability*
**Managing pain**	1	2.139	109.97	1.984	0.159
	2	1.807	109.03	6.304	0.012
	3	0.940	108.09	3.056	0.080
	4	1.642	107.16	0.171	0.680
	5	1.445	109.97	0.682	0.409
	6	2.684	107.16	5.401	0.020
**Movement**	7	-0.332	109.03	0.175	0.676
	8	0.036	109.03	0.630	0.427
	9	-0.334	108.09	0.177	0.674
	10	1.290	109.97	1.880	0.170
	11	-0.423	109.97	2.324	0.127
**Feelings**	12	-0.568	109.03	1.418	0.234
	13	1.325	109.97	0.163	0.686
	14	0.677	109.03	1.382	0.240
	15	1.168	109.03	0.022	0.883
**Arthritis process**	16	3.143	109.97	8.008	0.005
	17	1.595	107.16	0.304	0.581
	18	2.196	106.22	6.629	0.010
	19	-0.808	108.09	0.644	0.422
	20	-1.110	108.09	2.137	0.144
	21	-1.617	109.97	5.172	0.023
	22	2.191	108.09	1.589	0.207
**Treatments from health professionals**	23	-0.807	109.97	0.411	0.522
	24	0.066	109.97	0.423	0.515
	25	2.549	109.97	6.232	0.013
	26	-2.439	109.97	7.108	0.008
	27	-1.066	109.97	2.604	0.107
	28	-0.443	109.03	0.120	0.729
	29	-0.074	109.97	0.110	0.740
**Self-help measures**	30	0.692	109.03	0.010	0.920
	31	-0.363	109.97	0.419	0.517
	32	-1.557	109.03	6.869	0.009
	33	-0.704	109.03	6.448	0.011
	34	-0.102	109.97	1.302	0.254
	35	-0.958	109.97	1.509	0.219
**Support systems**	36	-0.098	109.97	0.227	0.633
	37	0.109	109.97	0.014	0.907
	38	1.829	109.97	0.167	0.682
	39	0.155	109.97	0.116	0.733

*Fit to the model: Bonferroni adjusted Chi square P >0.002.

Supplementary Table 3. Item fit statistics for the 7 domains, Italian ENAT (I-ENAT).

	Fit Residuals	Degrees of freedom	Chi Square	Probability
Managing pain	0.326	90.970	0.052	0.820
Movement	0.874	93.430	0.068	0.795
Feelings	1.269	95.070	0.043	0.835
Arthritis process	0.733	90.150	0.530	0.467
Treatments from health professionals	-0.428	95.070	0.841	0.359
Self-help measures	-0.657	93.430	1.173	0.279
Support systems	0.495	95.890	0.654	0.419
Fit to the model	*Within +/-2.500*			*>0.050*

Supplementary Table 4. Association of Rasch-transformed scores of the I-ENAT with continuous variables (Spearman's correlation).

		Total I-ENAT	Managing pain	Movement	Feelings	Arthritis process	Treatments from health professionals	Self-help measures	Support systems systems
**Age**	Correlation Coefficient	-0.011	-0.059	0.04	0.078	0.025	0.069	-0.038	-0.001
	p value		0.666	0.401	0.796	0.456	0.68	0.988	0.911
**Disease duration**	Correlation Coefficient	-0.127	-0.071	-0.003	-0.024	-0.138	-0.028	-0.111	-0.095
	p value	0.194	0.455	0.978	0.797	0.145	0.766	0.231	0.304
**School leaving age. years**	Correlation Coefficient	-0.148	-0.015	-0.14	-0.056	-0.083	-0.158	-0.095	-0.205*
	p value	0.129	0.871	0.133	0.547	0.382	0.085	0.304	0.025
**C-reactive protein**	Correlation Coefficient	0.065	0.141	0.04	-0.123	0.093	0.079	-0.07	0.003
	p value	0.743	0.449	0.826	0.496	0.617	0.661	0.7	0.985
**Erythrocyte sedimentation rate**	Correlation Coefficient	0.242*	0.220*	0.143	0.262*	0.06	0.266*	0.109	0.212*
	p value	0.017	0.024	0.141	0.006	0.549	0.005	0.265	0.027
**Swollen joint count**	Correlation Coefficient	0.017	0.116	0.103	0.054	-0.106	0.001	-0.073	0.124
	p value	0.868	0.233	0.285	0.569	0.278	0.992	0.446	0.193
**Tender joint count**	Correlation Coefficient	-0.122	-0.079	-0.02	-0.125	-0.208*	-0.091	-0.188	-0.006
	p value	0.231	0.42	0.838	0.19	0.033	0.342	0.051	0.952
**Patient - VAS, mm**	Correlation Coefficient	0.326*	0.205*	0.301*	0.201*	0.222*	0.209*	0.235*	0.300*
	p value	0.001	0.029	0.001	0.028	0.018	0.022	0.01	0.001
**28-joint disease activity score (CRP)**	Correlation Coefficient	0.07	0.126	0.141	0.016	-0.073	0.067	-0.026	0.136
	p value	0.494	0.197	0.144	0.868	0.456	0.488	0.789	0.151
**28-joint disease activity score (ESR)**	Correlation Coefficient	0.136	0.196	0.157	0.083	-0.095	0.118	0.043	0.188
	p value	0.193	0.05	0.115	0.404	0.354	0.232	0.67	0.056
				**Patient-reported outcomes**					
**HAQ**	Correlation Coefficient	0.286*	0.096	0.400*	0.287*	0.228*	0.204*	0.164	0.246*
	p value	0.003	0.312	<0.001	0.002	0.015	0.026	0.076	0.007
**Severity**	Correlation Coefficient	0.157	0.058	0.250*	0.131	0.207*	0.091	0.098	0.054
	p value	0.107	0.545	0.007	0.159	0.028	0.325	0.294	0.562
**Health**	Correlation Coefficient	0.212*	0.088	0.298*	0.250*	0.18	0.206*	0.131	0.221*
	p value	0.028	0.35	0.001	0.006	0.056	0.025	0.159	0.015

*significant associations (p value <0.05).

IQR interquartile range. CRP C-reactive protein. ESR erythrocyte sedimentation rate. VAS Visual Analogic Score. HAQ Heath assessment questionnaire.

Supplementary Table 5. Association of Rasch-transformed I-ENAT scores with categorical variables (Kruskal-Wallis test and post-hoc tests), all values reported as medians and interquartile range of the I-ENAT scores.

	Total I-ENAT	Managing pain	Movement	Feelings	Arthritis process	Treatments from health professionals	Self-help measures	Support systems
**Gender**								
**Males**	54.3 (44.5;72.9)	11.5 (7.9;13)	6.2 (3.7;10.6)	6.3 (4.9;7.2)	12.4 (5.6;17)	6.5 (5.1;15.9)	7.1 (3.7;13)	5.3 (4.6;8.9)
**Females**	86.2 (65.3;106.4)	13.5 (10.3;15.7)	9.5 (7.4;12.1)	9.2 (5.9;11.7)	16.2 (12.4;20.8)	13.4 (10;17.7)	13.7 (4.9;17.7)	9.4 (6.2;10.4)
**p value**	0.003	0.014	0.038	0.007	0.042	0.023	0.02	0.009
**Smoke**								
**Non smoker**	83.2 (66.4;99.6)	13.5 (10.3;15.4)	9.5 (6.8;11.8)	8.3 (5.9;10.3)	14.7 (10.6;20.8)	13.4 (7.5;17.7)	13.7 (4.9;16.3)	8.4 (5.3;10.4)
**Smoker**	86.5 (51.9;114.3)	13.5 (9.5;15.4)	8.5 (4.7;14.7)	9.2 (7.4;16)	17 (12;21.6)	13.4 (6.2;24)	12.2 (4.3;17.7)	7.2 (5.3;10.4)
**Former smoker**	67.3 (45.4;97.8)	11.5 (8.4;14.7)	9 (4.5;10.9)	6.6 (5.8;9.2)	14.7 (10.6;20.8)	11 (5.5;17.7)	12.2 (3.7;18.6)	6.2 (4.6;9.4)
**p value**	0.271	0.22	0.155	0.879	0.983	0.595	0.648	0.116
**Social Status**								
**Married/domestic partner, n (%)**	82.8 (56.8;100.3)	13.5 (9.5;15.4)	9.5 (6.4;11.8)	8.3 (5.9;10.3)	15.4 (10.6;20.8)	13.4 (7.2;17.7)	13.7 (4.3;17)	8.4 (5.3;10.4)
**Widow/widower**	63.3 (63.3;63.3)	8.6 (8.6;8.6)	7.6 (7.6;7.6)	9.2 (9.2;9.2)	12.4 (12.4;12.4)	10 (10;10)	7.1 (7.1;7.1)	8.4 (8.4;8.4)
**Divorced**	83.5 (73.8;87.9)	13.5 (9.5;14.5)	8.5 (6.2;10.6)	9.2 (5.9;10.3)	13.9 (12.4;17)	11.9 (10;14.8)	13.7 (7.1;16.3)	6.2 (5.3;10.4)
**Not married**	70.9 (57;101.6)	14.5 (10.8;16.4)	10.1 (6.2;15.3)	6.6 (5.2;9.2)	17 (11.5;19.8)	11.9 (5.4;18.1)	12.2 (6.6;18.6)	8.9 (6;10.7)
**p value**	0.873	0.577	0.818	0.819	0.939	0.558	0.961	0.885
**Employment**								
**Full-time employement**	72 (47.3;94.9)	11.5 (7.7;15.9)	8.5 (4.8;12.8)	6.6 (4.7;11)	13.9 (11.5;17.9)	10 (5.1;16.2)	12.2 (4.9;13.7)	6.2 (5.3;9.9)
**Housewife**	82.8 (67.6;101.7)	13.5 (8.6;19.7)	9.5 (6.2;14.7)	7.4 (5.9;9.2)	15.4 (10.6;18.8)	17.7 (13.4;28)	16.3 (13.7;19.1)	9.4 (8.4;10.4)
**Retired**	73.8 (52.4;95.8)	13.5 (9.5;14.3)	9.5 (8.1;10.6)	6.6 (5.6;9.2)	15.4 (12.4;20.8)	13.4 (8.7;17)	12.2 (3.8;16.3)	6.2 (4;9.4)
**Part-time employement**	149.4 (149.4;149.4)	17.4 (17.4;17.4)	20 (20;20)	16 (16;16)	28 (28;28)	28 (28;28)	24 (24;24)	16 (16;16)
**Invalid**	86.4 (69.6;102.6)	13.5 (11;15.4)	10.1 (7.6;11.8)	9.2 (7.4;11)	17 (12.4;20.8)	13.4 (10;19.3)	13.7 (7.1;16.3)	9.4 (6.2;10.4)
**Unemployed**	110.1 (52.2;123.3)	16.4 (14.5;19.7)	11.8 (6.8;16.9)	10.3 (5.9;11.7)	20.8 (8.8;28)	17.7 (10;21.2)	19.1 (4.9;21.2)	10.4 (9.4;12.6)
**Student**	41.3 (38;44.5)	9 (7.7;10.2)	4.4 (4.1;4.8)	3.8 (3;4.5)	10.6 (10.6;10.6)	4.6 (4.1;5)	4.7 (3.5;5.9)	4.3 (4.2;4.5)
**Other**	108.9 (108.9;108.9)	15.5 (15;15.9)	12.7 (11.6;13.7)	11 (8.4;13.5)	5.6 (5.6;5.6)	22.1 (19.2;25.1)	24 (24;24)	16 (16;16)
**p value**	0.123	0.165	0.271	0.113	0.451	0.034	0.046	0.007
**Post-hoc tests**						No significant difference	No significant Difference	No significant difference
**Housewife**								
**Non-housewife**	73.8 (49.6;99.6)	13.5 (9.5;15.4)	8.5 (6;11.8)	6.6 (5.3;10.3)	15.4 (10.6;20.8)	13.4 (5.5;17.7)	12.2 (4.3;17.7)	7.2 (5.3;10.4)
**Housewife**	86.4 (69.6;102.6)	13.5 (11;15.4)	10.1 (7.6;11.8)	9.2 (7.4;11)	17 (12.4;20.8)	13.4 (10;19.3)	13.7 (7.1;16.3)	9.4 (6.2;10.4)
**p value**	0.214	0.713	0.292	0.051	0.477	0.296	0.829	0.197
**Retired**								
**Non-retired**	83.7 (58.2;105.1)	13.5 (9.5;16.4)	9.5 (6.2;13.1)	8.3 (5.9;11.7)	13.9 (10.6;20.8)	13.4 (6.2;19.8)	13.7 (4.9;16.3)	8.9 (5.3;10.4)
**Retired**	73.8 (52.4;95.8)	13.5 (9.5;14.3)	9.5 (8.1;10.6)	6.6 (5.6;9.2)	15.4 (12.4;20.8)	13.4 (8.7;17)	12.2 (3.8;16.3)	6.2 (4;9.4)
**p value**	0.4	0.403	0.964	0.18	0.737	0.804	0.702	0.037
**Full-time employement**								
**Non-full-time**	84.5 (59.5;103.9)	13.5 (9.5;15.4)	9.5 (7.2;11.8)	8.3 (5.9;10.3)	15.4 (10.6;20.8)	13.4 (10;17.7)	13.7 (4.3;19.1)	8.4 (5.3;10.4)
**Full-time employement**	72 (47.3;94.9)	11.5 (7.7;15.9)	8.5 (4.8;12.8)	6.6 (4.7;11)	13.9 (11.5;17.9)	10 (5.1;16.2)	12.2 (4.9;13.7)	6.2 (5.3;9.9)
**p value**	0.243	0.447	0.245	0.36	0.41	0.029	0.166	0.283
**Concomitant**								

**comorbidity**								
**No**	82.8 (54;98.2)	13.5 (9.5;15.4)	9.5 (6.2;11.8)	7.4 (5.5;10)	15.4 (10.6;20.8)	13.4 (5.7;17.7)	12.2 (4.9;16.3)	8.4 (5.3;10.4)
**Yes**	82.5 (58.7;102.5)	12.5 (9.5;15.4)	9 (6.2;11.8)	8.3 (5.9;10.3)	15.4 (12.4;20.8)	13.4 (10;17.7)	13.7 (4.3;18.1)	9.4 (5.3;10.4)
**p value**	0.575	0.522	0.949	0.343	0.688	0.727	0.666	0.65
**Patient-reported Outcomes**								
**Willing to receive information**								
**No**	52.2 (38.5;94.7)	9.5 (7.1;12)	6.2 (4;9.5)	5.9 (5;8.8)	8.1 (3.2;13.9)	6.2 (4.8;13.4)	7.1 (3.5;13.7)	6.2 (5;9.4)
**Yes**	85.6 (64;103.9)	13.5 (10.5;15.9)	10.1 (7.6;12.8)	8.3 (5.9;10.3)	17 (12.4;20.8)	13.4 (10;17.7)	13.7 (4.9;16.3)	8.4 (5.3;10.4)
**p value**	0.007	<0.001	0.012	0.115	<0.001	0.005	0.114	0.057
**How much information**								
**1**	43 (40.8;44)	8.6 (8.2;12)	6.2 (3.1;7.4)	6.6 (6.6;6.6)	4.1 (3.8;9)	4.4 (2.2;4.8)	3.8 (3.5;5.5)	4.6 (4.6;5.4)
**2**	43.4 (28.6;69)	8.6 (6.7;11.3)	7.2 (3.7;9.3)	4.7 (3.8;7)	6.5 (2.4;12.4)	5.9 (4.1;11.9)	3.5 (3.5;10.2)	5.8 (3;8.1)
**3**	70.1 (64.4;95)	11.5 (10.5;14.5)	9.5 (6.2;10.6)	7.4 (5.9;9.2)	17 (12.4;18.8)	14.8 (6.2;17.7)	7.1 (4.3;13.7)	8.4 (6.2;10.4)
**4**	88.4 (71.3;109.2)	14.5 (11;16.4)	10.6 (7;14.7)	9.2 (5.9;10.7)	17 (12.4;20.8)	13.4 (10;19.8)	13.7 (7.1;18.4)	9.4 (5.3;10.4)
**p value**	<0.001	0.001	0.015	0.013	<0.001	0.001	<0.001	0.009
**Post-hoc tests**	Categories 1 and 4. p=0.039; 2 and 4. p<0.001	Categories 2 and 4. p=0.001	Categories 2 and 4. p=0.031	Categories 2 and 4. p=0.008	Categories 2 and 3. p=0.007; 2 and 4. p<0.001	Categories 1 and 4. p=0.038; 2 and 4. p=0.014	Categories 2 and 4. p=0.001	Categories 2 and 4. p=0.018
**Flare**								
**Yes**	86.6 (70.5;103.3)	13.5 (10.3;15.7)	10.6 (6.8;14.7)	9.2 (5.9;10.3)	16.2 (12.4;21.6)	15.5 (10;19.3)	13.7 (7.1;16.3)	8.4 (5.8;10.4)
**No**	70.9 (49.6;96.8)	12.5 (9.5;15.4)	8.5 (5.7;10.6)	7.4 (5.3;10)	13.9 (9.3;18.8)	11.9 (5.5;16.2)	12.2 (4.3;16.3)	7.8 (4.6;10.4)
**p value**	0.075	0.423	0.115	0.325	0.207	0.023	0.408	0.162
**Health**								
**1**	66.1 (63.9;86.7)	9.5 (8.6;11.5)	4.2 (3.7;6.2)	5.3 (4.3;8.4)	13.9 (12.4;15.4)	9.7 (7.5;13)	13 (6.3;17.8)	6.3 (5.3;8.9)
**2**	68.7 (46.9;86.3)	11.5 (8.6;15.4)	7.6 (4.9;10.6)	6.6 (4.7;9.2)	12.4 (8.8;17)	10 (5.5;14.1)	7.1 (4.3;13.3)	5.3 (4.3;8.9)
**3**	95 (69.6;111.2)	13.5 (11.5;15.4)	10.1 (7.8;12.8)	8.3 (5.9;9.8)	17 (12.4;23.8)	16.2 (12.7;22.6)	14.4 (7.1;19.1)	9.4 (7.3;10.9)
**4**	85.7 (62;96.2)	13.5 (9.5;16.4)	10.6 (7.2;14.7)	9.2 (5.9;11.7)	13.9 (12.4;20.8)	13.4 (10;17.7)	13 (6.4;16.3)	8.4 (6.2;10.4)
**p value**	0.024	0.284	0.003	0.059	0.057	0.004	0.049	0.004
**Post-hoc tests**	Categories 2 and 3. p=0.024		Categories 1 and 3. p=0.037; 1 and 4 p=0.030			Categories 2 and 3. p=0.003	Categories 2 and 3. p=0.031	Categories 2 and 3. p=0.031
**Severity**								
**1**	66.1 (44.3;90.4)	12 (8.4;15.4)	6.2 (5.1;8.5)	7 (4.3;9)	13.9 (5.2;17)	11.9 (6;14.8)	12.2 (4.3;15.7)	8.3 (4.2;10.2)
**2**	72 (54;99.9)	13.5 (9.5;15.4)	8.5 (6.5;10.6)	8.3 (5.3;9.2)	12.4 (8.8;20.8)	13.4 (5.5;18.6)	13 (4.9;16.3)	8.4 (5.3;10.4)
**3**	85.2 (57.5;108.3)	13.5 (9.5;16.4)	9.5 (6.2;14.7)	7.4 (5.9;11.7)	17 (12.4;20.8)	13.4 (7.5;17.7)	13 (4.3;18.8)	8.4 (6.2;10.4)
**4**	88.9 (70.9;96.9)	13.5 (10.5;15.4)	10.6 (10.6;14.7)	9.2 (5.9;10.3)	17 (13.9;20.8)	16.2 (11.9;17.7)	13.7 (12.2;16.3)	6.2 (4.6;10.4)
**p value**	0.441	0.922	0.056	0.558	0.189	0.693	0.742	0.627
